# Supplementary material for: The interactome of intact mitochondria by cross-linking mass spectrometry provides evidence for coexisting respiratory supercomplexes
Source: Mol Cell Proteomics. 2017 Dec 8;17(2):216–32. doi: 10.1074/mcp.RA117.000470 (PMC5795388; doi:10.1074/mcp.RA117.000470)
Supplement: Supplemental Data [file supp_RA117.000470_133922_0_supp_23978_fzffwf.zip › spectra_annotation/mito_DR_spectra_annotation/460-1-1-1-5-1.pdf]

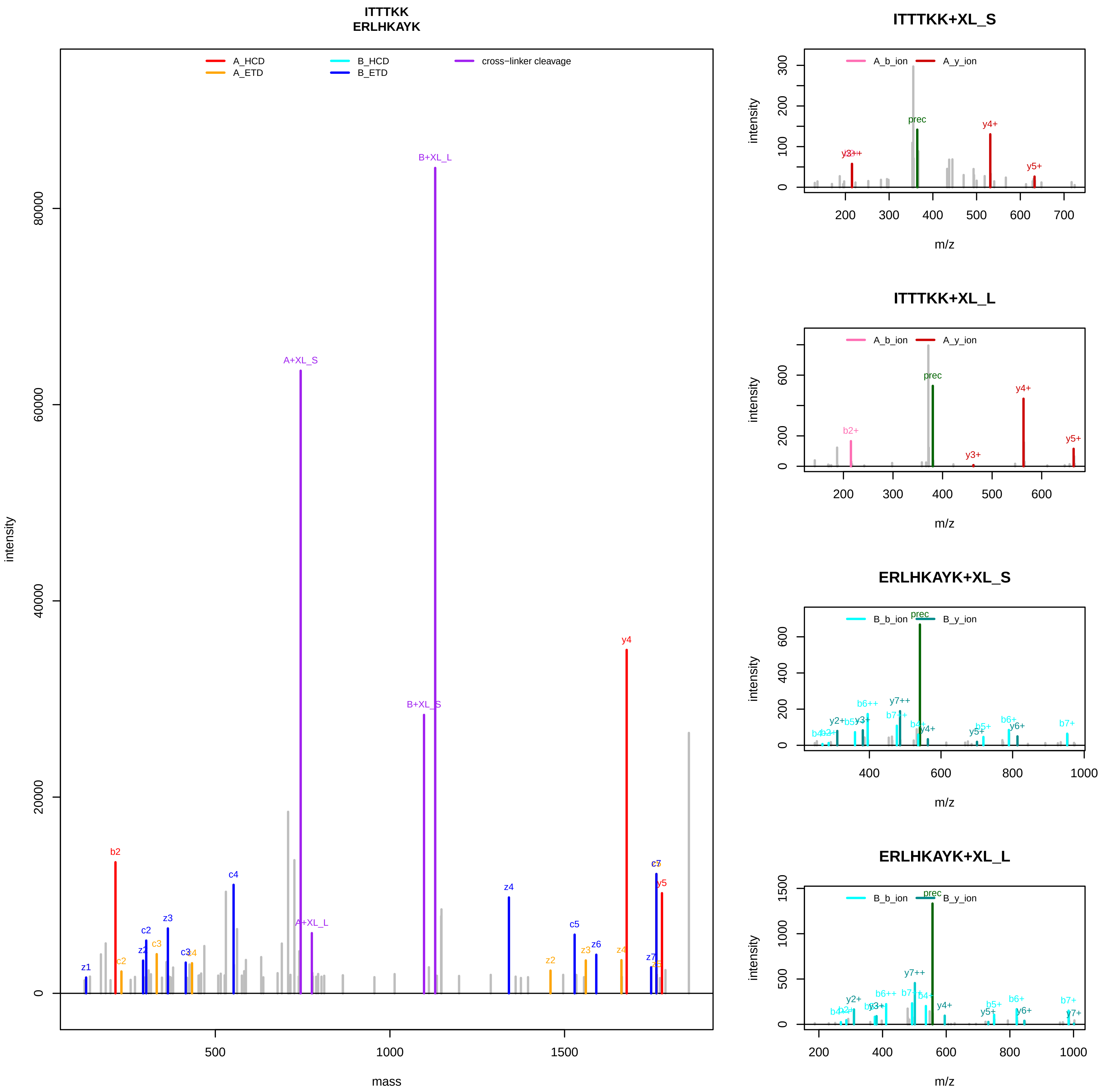

ITTTKK
ERLHKAYK
A_HCD
A_ETD
B_HCD
B_ETD
cross−linker cleavage
intensity
mass
0
20000
40000
60000
80000
500
1000
1500
A+XL_S
B+XL_L
B+XL_S
A+XL_L
y4
b2
c4
z4
c5
z6
z3
c2
c3
z1
z2
c7
y5
z7
ITTTKK+XL_S
A_b_ion
A_y_ion
prec
y4+
y3++
y5+
m/z
ITTTKK+XL_L
b2+
y3+
ERLHKAYK+XL_S
B_b_ion
B_y_ion
y7++
b6++
b7++
y2+
y3+
b4+
y4+
y5+
b5+
b6+
y6+
b7+
ERLHKAYK+XL_L
y4+
y7+
